# Editorial: Neuroelectronics: towards symbiosis of neuronal systems and emerging electronics

**DOI:** 10.3389/fnins.2023.1227798

**Published:** 2023-06-07

**Authors:** Alexey N. Mikhaylov, Sergey A. Shchanikov, Vyacheslav A. Demin, Valeri A. Makarov, Victor B. Kazantsev

**Affiliations:** ^1^Laboratory of Stochastic Multistable Systems, Department of Neurotechnologies, Lobachevsky State University of Nizhny Novgorod, Nizhny Novgorod, Russia; ^2^Murom Institute, Vladimir State University, Murom, Russia; ^3^Department of Radio Engineering and Cybernetics, Laboratory of Neurobiomorphic Technologies, Moscow Institute of Physics and Technology, Dolgoprudny, Russia; ^4^Kurchatov Complex for NBICS Nature-inspired Technologies, Kurchatov Institute, Moscow, Russia; ^5^Interdisciplinary Mathematics Institute, Complutense University of Madrid, Madrid, Spain; ^6^Institute of Fundamental Medicine, Privolzhsky Research Medical University, Nizhny Novgorod, Russia

**Keywords:** neuroelectronics, neuromorphic system, neurohybrid system, memristor, neural interface, artificial intelligence, neuroprosthetics, neurosensing

## Introduction

Studies in neuromorphic and neurohybrid systems currently represent one of the most exciting and intriguing multidisciplinary trends in modern science and technology. They integrate the fields of neuroscience, electronics, physics, and mathematics. Recent progress in building artificial neurons and neural networks based on microelectronic devices and memristive crossbars stimulates a qualitative leap toward general artificial intelligence (AI). In this respect, neuroelectronics can be defined as a synthesis of analog and digital solutions for a wide range of computational tasks motivated by living neural systems. Analog neuromorphic systems based on standard or memristive components are among the peculiarities of this approach. They can significantly improve throughput and energy efficiency compared to AI accelerators based on digital components. Such systems mimic computational features of biological neural networks, which can solve ill-understood tasks (often described as “cognitive”) known to be either intractable by traditional AI or highly time-consuming. In addition, neuroelectronic solutions can be integrated with the brain or living neuronal circuits and form neurohybrid systems. Such systems can take advantage of complex molecular mechanisms of biological cells (e.g., memory and adaptation) and support fast computations carried out by the artificial part of the tandem. The symbiosis of natural and artificial systems may also make it possible to develop new learning approaches for neuromorphic devices where a network of living neurons acts as a “teacher.”

A strategic question from both fundamental and applied points of view is the involvement of living neural networks in synthetic information processing. It implies the implementation of computation and learning in artificial networks that are shaped through their interaction with living systems, eventually implementing specific brain functions (replacing damaged neural circuits or enhancing their functionalities). Steps toward this authentically hybrid approach based on bi-directional interaction between synthetic and biological systems can lead to significant benefits: They can lead to a technological breakthrough in such applications of neurohybrid systems as neuroimplants and neuroprosthetics, as well as the emergence of a new class of AI, including the systems using living cells as an element base for computing.

The main goal of this Research Topic was to stimulate interaction and discussion between different aspects of this “authentically hybrid” challenge, including—but not limited to—electronics and neurotechnologies. The former incorporates investigating new materials, emerging memory devices, and brain-like computing systems. At the same time, the latter covers cellular and network mechanisms of brain function, adaptation, and plasticity in neural networks, biocompatible materials, recording systems, etc. While this Research Topic seeks to bring together an extensive range of communities, it maintains a sharp focus on functional bio-interfacing.

## Toward symbiosis of neuronal systems and emerging electronics

In a review, Burelo et al. analyze in a unified manner previously published results to demonstrate the capabilities of neuromorphic technologies and systems based on standard electronic components for online and real-time processing of brain signals. A spiking neural network implemented on a special neuromorphic processor with analog silicon neurons is used to analyze interictal high-frequency oscillations (HFO) detected in EEG recordings as biomarkers of epileptogenesis and predisposition to seizures. The authors show that a neuromorphic approach to HFO detection can be applied to recordings of various modalities (intracranial electroencephalography, electrocorticography, and scalp electroencephalography). Real-time HFO detection may improve the outcome of epileptic seizures. Using a neuromorphic processor for non-invasive therapy monitoring might allow for more effective medication strategies to achieve seizure control.

The paper by Mikhailova et al. reports the development and application of a neural interface to compensate muscle hypertonus via stimulation by bio-plausible patterns. The neural interface includes a neuroport based on a DAC and stimulator and a neuroterminal used for neurostimulation of oscillator motifs on a single-board computer. The approach is demonstrated on five volunteers by stimulating the antagonist muscle or nerve and recording muscle force with and without antagonist stimulation. The optimal modes of stimulation are determined based on the subjective discomfort rate. The authors plan to continue this research to organize a feedback to adjust stimulation patterns based on the EMG data.

The use of a neural interface for studying neurocognitive processes is clearly demonstrated by Radchenko et al. in the example of music perception, which is increasingly used as a therapeutic tool in rehabilitation medicine and psychophysiology. Twenty volunteers with no musical education took part in a study of the influence of the music's temporal organization, which differed in tempo (fast vs. slow) and meter (duple vs. triple). It is shown that the N200 wave occurs significantly faster for stimuli with duple meter and fast tempo and is the slowest for stimuli with triple meter and fast pace.

The capabilities of emerging electronic components in creating adaptive prosthetic devices are presented by Masaev et al. on the example of organic memristors. These devices are capable of self-learning according to bio-plausible spike-timing-dependent plasticity and are used for the first time to organize the electrical activity of the walking pattern generated by the central pattern generator (CPG). Unlike standard digital generator circuits with manually adjusted parameters, simple self-organizing circuits based on memristors are an essential step forward to the hardware implementation of adaptive CPGs.

Andreeva et al. presented various functionalities of memristors based on CMOS-compatible materials and structures. Depending on the composition of nanolayers in the Pt/TiO_2_/Al_2_O_3_/Pt device and on the structure of the titanium oxide layer, the authors demonstrate a wide range of resistance change, which is responsible for synaptic behavior. Replacing an inert electrode with an active one makes obtaining neuronal activity in the same device possible due to a pronounced N-shaped feature of *I-V* characteristics. The proposed approach makes it possible to implement artificial neural network algorithms at the material level and simplifies neuromorphic circuits while maintaining all benefits of neuromorphic architectures.

## Conclusion

This current Research Topic is the first attempt to bring together cutting-edge research in neuroelectronics. This discipline includes new brain-like electronic systems that mimic the human brain and nervous system and new approaches to integrating these systems with biological ones as part of open-loop or closed-loop interfaces for the recording and exciting biological signals. The maturation of new technologies and electronic components based on memristors will provide unique opportunities for achieving energy efficiency and compactness of such systems and, in the case of biocompatible materials, to move to a symbiosis of electronic and biological systems at all levels of organization—from the molecular level to the whole organism.

## Author contributions

All authors gave substantial contribution to the development of this work equally, drafting and revising it critically, and approved its final version for publication.

